# Polyfunctional T-Cell Responses Are Disrupted by the Ovarian Cancer Ascites Environment and Only Partially Restored by Clinically Relevant Cytokines

**DOI:** 10.1371/journal.pone.0015625

**Published:** 2010-12-22

**Authors:** Eric Tran, Julie S. Nielsen, Darin A. Wick, Alvin V. Ng, Lisa D. S. Johnson, Nancy J. Nesslinger, Elissa McMurtrie, John R. Webb, Brad H. Nelson

**Affiliations:** 1 Department of Microbiology and Biochemistry, University of Victoria, Victoria, British Columbia, Canada; 2 Trev and Joyce Deeley Research Centre, British Columbia Cancer Agency, Victoria, British Columbia, Canada; 3 Department of Medical Genetics, University of British Columbia, Vancouver, British Columbia, Canada; 4 BC Cancer Agency, Victoria, British Columbia, Canada; Florida International University, United States of America

## Abstract

**Background:**

Host T-cell responses are associated with favorable outcomes in epithelial ovarian cancer (EOC), but it remains unclear how best to promote these responses in patients. Toward this goal, we evaluated a panel of clinically relevant cytokines for the ability to enhance multiple T-cell effector functions (polyfunctionality) in the native tumor environment.

**Methodology/Principal Findings:**

Experiments were performed with resident CD8+ and CD4+ T cells in bulk ascites cell preparations from high-grade serous EOC patients. T cells were stimulated with α-CD3 in the presence of 100% autologous ascites fluid with or without exogenous IL-2, IL-12, IL-18 or IL-21, alone or in combination. T-cell proliferation (Ki-67) and function (IFN-γ, TNF-α, IL-2, CCL4, and CD107a expression) were assessed by multi-parameter flow cytometry. In parallel, 27 cytokines were measured in culture supernatants. While ascites fluid had variable effects on CD8+ and CD4+ T-cell proliferation, it inhibited T-cell function in most patient samples, with CD107a, IFN-γ, and CCL4 showing the greatest inhibition. This was accompanied by reduced levels of IL-1β, IL-1ra, IL-9, IL-17, G-CSF, GM-CSF, Mip-1α, PDGF-bb, and bFGF in culture supernatants. T-cell proliferation was enhanced by exogenous IL-2, but other T-cell functions were largely unaffected by single cytokines. The combination of IL-2 with cytokines engaging complementary signaling pathways, in particular IL-12 and IL-18, enhanced expression of IFN-γ, TNF-α, and CCL4 in all patient samples by promoting polyfunctional T-cell responses. Despite this, other functional parameters generally remained inhibited.

**Conclusions/Significance:**

The EOC ascites environment disrupts multiple T-cell functions, and exogenous cytokines engaging diverse signaling pathways only partially reverse these effects. Our results may explain the limited efficacy of cytokine therapies for EOC to date. Full restoration of T-cell function will require activation of signaling pathways beyond those engaged by IL-2, IL-12, IL-18, and IL-21.

## Introduction

Numerous studies in the past decade have shown that the immune system influences clinical outcomes in patients with high-grade serous epithelial ovarian cancer (hereafter referred to as EOC). Specifically, the presence of tumor-infiltrating CD8+ T cells is associated with prolonged disease-free and overall survival [1], [2]. Moreover, elevated numbers of CD56+ T cells in the ascites compartment are correlated with increased platinum sensitivity [3]. More recently, ascites fluid containing elevated levels of IL-17 (a cytokine predominately produced by Th17 and other effector T cells [4]) was correlated with increased overall survival [5]. Together, these studies suggest that enhancing the endogenous T-cell response to EOC may be of clinical benefit to patients. However, the ovarian tumor environment contains many immunosuppressive cell types and factors that can oppose anti-tumor T-cell responses. Accordingly, elevated numbers of tumor-associated regulatory T cells (Tregs) and macrophages are associated with poor survival in EOC [1], [2]. Many soluble immunosuppressive factors are also found in ascites, including IL-10, TGF-β, VEGF, B7-H1/PD-L1, B7-H4, SDF-1, EBAG9/RCAS1, Fas ligand, and soluble IL-2 receptor [1], [2].

The heterogeneity of immunosuppressive mechanisms in EOC presents a formidable challenge for immunotherapy, as it suggests that multiple immunosuppressive mechanisms might need to be reversed to restore T-cell function. For example, Treg depletion is being evaluated as a means to enhance immunity against several human cancers [6], including EOC [7], but at best would only reverse one mechanism of immunosuppression, leaving other immunological barriers intact. Rather than attempt to disable these barriers one by one, a more pragmatic clinical approach would be to deliver factors that can broadly override immunosuppressive barriers in the tumor environment. Thus, identifying the signals or conditions that promote T-cell responses in the EOC environment may lead to more effective immunotherapeutic strategies.

To this end, we previously developed a mouse model of EOC that allows functional assessment of CD8+ T cell responses in the ovarian tumor environment [8]. Specifically, we engineered a murine EOC tumor line to express a CD8+ T cell epitope from the model antigen ovalbumin. Mice bearing advanced, widely disseminated tumors with extensive ascites were treated by adoptive transfer of CD8+ T cells specific for the ovalbumin epitope. Remarkably, adoptively transferred CD8+ T cells underwent vigorous proliferation not only in lymph nodes and peripheral blood, but also in the ascites and tumor compartments. Indeed, at the peak of the response, the transferred T cells constituted up to 40% of CD8+ T cells in peripheral blood, and up to 96% of CD8+ T cells in ascites. This profound proliferative response was followed by rapid and near-complete tumor regression in the majority of animals, demonstrating that the T cells were functionally active. Importantly, the proliferation and anti-tumor activity of the transferred CD8+ T cells was entirely dependent on IL-2/IL-15 signaling, as demonstrated by genetic disruption of the IL-2 receptor alpha (CD25) or beta (CD122) subunits [8]. Thus, even in the setting of advanced ovarian tumors, CD8+ T cells mounted potent anti-tumor responses provided that IL-2/IL-15 signaling pathways were intact.

The foregoing results raised the issue of whether cytokines such as IL-2 can similarly over-ride the immunosuppressive effects of ascites in human EOC. Previous *in vitro* studies with human EOC samples have shown that IL-2 can partially restore lymphokine-activated killer (LAK) cell cytotoxicity in the presence of 50% ascites fluid, while the combination of IL-2 with TCR stimulation [9] or IL-12 [10], [11] fully restored LAK cytotoxicity. However, use of 50% ascites fluid alone does not fully recapitulate the ovarian tumor environment, which also contains immunosuppressive cell types such as Tregs and MDSCs [1], [2]. Furthermore, LAKs are predominately comprised of NK cells, which may be affected differently by ascites compared to T cells. In addition, these past studies measured cytotoxicity by bulk LAK cell preparations and hence did not elucidate the effects of cytokines on different T cell subsets. Finally, *in vitro* cytotoxicity is only one aspect of T-cell function and does not always correlate with effective T-cell responses *in vivo* [12].

There is growing appreciation of the importance of polyfunctional T-cells (i.e., T cells that can simultaneously perform multiple functions) in effective immune responses [13]. Specifically, polyfunctional T-cell responses are associated with protective immunity after vaccination against smallpox (vaccinia virus) [14], yellow fever [15], tuberculosis [16], [17], and leishmaniasis [18]. Elevated numbers of polyfunctional T cells are also correlated with favorable outcomes in a variety of disease settings, including HIV/AIDS [19], [20], [21], [22], [23], [24], [25], [26], [27], [28], [29], hepatitis C [30], and lymphocytic choriomeningitis [31]. In the setting of cancer, several studies have found enhanced numbers of tumor-specific polyfunctional T cells in patients responding favorably to various forms of immunotherapy, including adoptive T-cell therapy, α-CTLA-4 antibody therapy, and tumor vaccines [32], [33], [34], [35], [36].

In the present study, we assessed for the first time the effects of the human EOC ascites environment on T-cell polyfunctionality. We also evaluated the ability of IL-2 and three other clinically relevant cytokines (IL-12, IL-18, and IL-21) to restore a broad range of T-cell functions. We found that ascites inhibited polyfunctional responses by both CD4+ and CD8+ T cells, with some functions showing greater inhibition than others. Furthermore, only a subset of T-cell functions was restored by exogenous cytokines delivered alone or in combination. Thus, full restoration of T-cell function in EOC will require activation of signaling pathways beyond those engaged by IL-2, IL-12, IL-18, and IL-21.

## Results

### EOC ascites disrupts multiple T-cell functions

Primary ascites specimens were collected from patients with high-grade serous EOC (Table 1). To model the native ascites tumor environment as closely as possible *in vitro*, we analyzed bulk ascites cell pellets, which in addition to CD8+ and CD4+ T cells, contained resident tumor cells, Tregs, B cells, NK cells, and CD14+ cells (Table 2). Moreover, cells were cultured in 100% autologous ascites fluid to include any soluble factors, such as TGF-β, IL-10, and VEGF (Table 2), that can impact T-cell function. Because of the paucity of well-defined T-cell antigens in ovarian cancer, we stimulated cultures with α-CD3 antibody, which should stimulate both effector and regulatory subsets. Thus, each patient's T cells were analyzed in the presence of the full complement of naturally occurring tumor cells, immunosuppressive cells, and soluble factors.

**Table 1:** Patient clinical characteristics.

Patient ID	Age at diagnosis	Pathologic diagnosis	Grade	FIGO Staging	TNM Staging
IROC008	70	Papillary serous carcinoma	3/3	4	pT3c, pN1
IROC028	61	Papillary serous carcinoma	3/3	3C	pT3c, NX, MX
IROC034	64	Papillary serous carcinoma	3/3	N/A	T3c
IROC036	60	Papillary serous carcinoma	3/3	N/A	T3c, NX, MX
IROC038	40	Papillary serous carcinoma	3/3	3B	pT3b

Median Age: 61; Mean Age: 59; Age Range: 40–70.

N/A, not assessed.

**Table 2:** Cellular composition and TGF-β, IL-10, and VEGF levels in the ascites compartment of EOC patients in this study.

Parameter	IROC Patient Sample				
	008	028^A^	034	036	038
Lymphocytes^B^	28.5	74.6	78.0	20.6	40.2
CD3+	19.8	59.0	58.0	11.9	21.9
CD3+CD4+	6.5	41.1	26.8	4.5	6.2
CD3+CD8+	12.4	16.9	28.1	6.6	13.0
CD3+CD56+	3.8	1.6	10.1	1.0	3.2
CD56+	2.7	12.0	9.7	3.0	4.6
Tregs^C^ (CD4+CD25+FoxP3+)	5.2	6.8	2.9	3.3	2.5
CD25+FoxP3+ in CD4+	12.4	12.1	12.1	6.8	10.7
B cells^C^ (CD19+)	8.1	1.4	6.5	4.5	5.0
CD14+	55.3	11.2	1.6	50.5	30.9
TGF-β (pg/ml)	6.3	ND^D^	33.3	ND	ND
IL-10 (pg/ml)	153.3	90.0	101.6	110.4	110.7
VEGF (pg/ml)	866.3	348.4	1304.4	2308.3	967.0

Cellular composition data is expressed as a percentage of total live cells. Cytokine data is expressed using the indicated units.

A Sample contained many large tumor rafts not detectable by FACS. Therefore, values are inflated since visual inspection and IHC staining shows this sample is comprised mainly of tumor cells.

B As determined by side and forward scatter.

C Tregs and B cells expressed as a percentage of total live lymphocytes.

D ND, not detectable.

**Figure 1:**
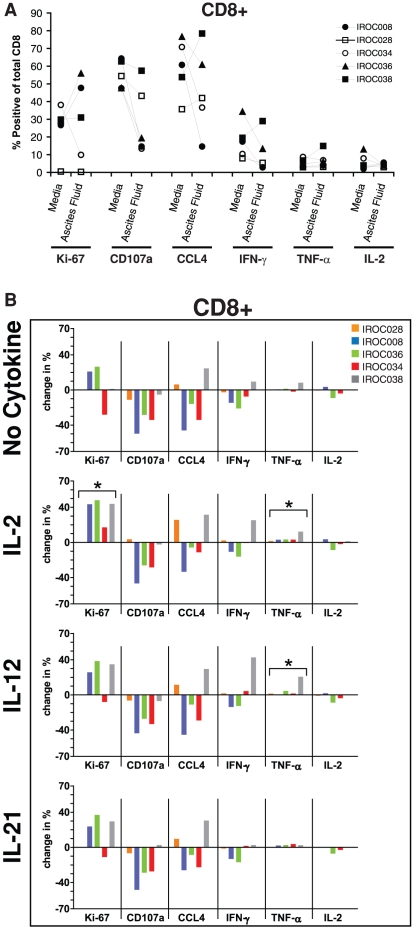
Impact of ascites fluid and IL-2, IL-12, and IL-21 on CD8+ T-cell proliferation and function. Bulk ascites cells were stimulated with plate bound α-CD3 in media or 100% autologous ascites fluid for 48 h. (**A**) Proliferation and function of CD8+ T cells in the presence of media or autologous ascites fluid (top panel) was assessed by measuring expression of Ki-67, CD107a, CCL4, IFN-γ, TNF-α, and IL-2 by flow cytometry. (**B**) Effect of IL-2 (second panel), IL-12 (third panel), and IL-21 (fourth panel) on CD8+ T-cell proliferation and various functions in the presence of autologous ascites fluid. Data has been normalized to α-CD3 stimulated cells in media (i.e., media values were subtracted from each stimulation condition). *There was a significant effect of the cytokine for enhancing the indicated function compared to cells stimulated in media (Wilcoxon matched pairs t test, p<0.05).

We recently demonstrated in a murine model of EOC that the ascites environment can be highly permissive to T-cell proliferation [8]. To see if this was also true in human EOC samples, we tested the effect of ascites fluid on T-cell proliferation. As seen in Fig. 1A, ascites fluid had highly variable effects on CD8+ T-cell proliferation (as measured by Ki-67 expression), ranging from strong inhibition (1/5 patients) to enhancement of proliferation (2/5 patients). In general, similar trends were seen for CD4+ T cells (data not shown). Thus, as suggested by our murine studies [8], the human EOC ascites environment is not universally suppressive and in some cases can enhance T-cell proliferation.

We next determined whether other T-cell functions were similarly affected by ovarian ascites. Flow cytometric analysis was used to assess five commonly used markers of T-cell function: IFN-γ, TNF-α, IL-2, CCL4, and CD107a [13], [14], [19], [20], [21]. The first four markers are cytokines involved in T-cell proliferation or effector function, whereas CD107a is expressed on the surface of T cells undergoing cellular degranulation and thus serves as a marker of cytotoxicity [13]. In 4/5 patient samples, ascites fluid caused a dramatic inhibition of CD107a expression by CD8+ T cells (Fig. 1A), which was accompanied by reduced levels of granzyme B in culture supernatants (Supp. Fig. S1A), indicating the ascites environment generally inhibits T-cell degranulation. CCL4 and IFN-γ expression were also inhibited by ascites in the majority of patient samples (Fig. 1A). T cells expressing IL-2 and TNF-α were relatively rare, and were minimally affected by ascites fluid in most cases (Fig. 1A). Similar results were seen with CD4+ T cells (data not shown). Intriguingly, the effects of ascites on T-cell proliferation and other T-cell functions were largely uncoupled. For example, with patient samples IROC008 and IROC036, ascites fluid enhanced both CD8+ and CD4+ T-cell proliferation but inhibited expression of CD107a, CCL4, and IFN-γ (Fig. 1A, and data not shown). Thus, ascites fluid has widely variable effects on T-cell functions between patient samples, in accord with the heterogeneous nature of EOC.

To further characterize the effects of ascites fluid on immune function, culture supernatants from the above experiments were evaluated by multiplex bead assays for expression of a broad panel of cytokines associated with immunological and inflammatory processes. Of 27 cytokines tested, nine (IL-1β, IL-1ra, IL-9, IL-17, G-CSF, GM-CSF, Mip-1α, PDGF-bb, and bFGF) were inhibited by ascites fluid compared to media in all 5 patient samples (Supp. Fig. S1B). Ascites fluid had variable effects on the remaining cytokines (data not shown). Although multiplex bead assays do not provide information regarding the cellular source(s) of cytokines, the results nonetheless underscore the broad effects of ascites fluid on the cytokine milieu in EOC.

Although a systematic evaluation of the myriad immunosuppressive factors in EOC [1], [2] was beyond the scope of this study, we did investigate the possible influence of Tregs, TGF-β, IL-10, and VEGF. The percentage of Tregs ranged from 2.5% to 6.8% of total cells but showed no significant correlation with the degree of inhibition of T-cell proliferation, or CD107a, CCL4, IFN-γ, TNF-α, and IL-2 expression; likewise, the levels of TGF-β, IL-10, and VEGF in ascites fluid did not significantly correlate with the degree of T-cell inhibition (Table 2; Pearson correlation p>0.05 for all comparisons). These findings underscore the complex immunobiology of the ovarian tumor environment in that the degree of immunosuppression did not correlate with these common immunosuppressive factors.

### Exogenous cytokines only partially restore T-cell function in the EOC ascites environment

In our mouse model of EOC, IL-2/IL-15 signaling was critical for CD8+ T-cell proliferation and function in ascites [8]. However, the preceding experiments revealed that the vast majority of T cells in EOC ascites did not express detectable levels of IL-2. This led us to speculate that the addition of IL-2 to cultures might restore T-cell proliferation and function. To test this, bulk ascites cells were stimulated with α-CD3 in the presence of 100% ascites fluid with or without exogenous IL-2, and T-cell proliferation and function were measured 48 h later. In 4/5 patient samples, IL-2 fully reversed the effects of ascites on T-cell proliferation and indeed induced responses greater than those seen in complete media (Fig. 1B). By contrast, IL-2 had little effect on the expression of CD107a, CCL4, IFN-γ, or IL-2 for most patient samples, although it caused a weak but statistically significant enhancement of TNF-α expression (Fig. 1B). Thus, IL-2 alone enhanced T-cell proliferation but, in general, had negligible effects on other functions.

We next asked if the engagement of other signaling pathways might better enhance T-cell function. Whereas IL-2 activates primarily the STAT5 pathway, the related cytokine IL-12 activates the STAT4 pathway, which has been shown to enhance T cell function in various physiological settings [37]. When added to EOC cultures, IL-12 alone had only minor effects on the proliferation or function of CD8+ T cells in most patient samples (Fig. 1B). We next evaluated IL-21, which predominantly activates the STAT1 and STAT3 pathways [38]. Similar to IL-12, IL-21 had only modest effects on T-cell proliferation or function (Fig. 1B). In general, similar trends were seen with CD4+ T cells (data not shown).

**Figure 2:**
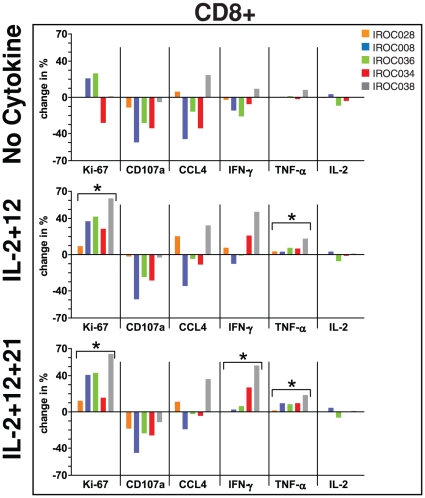
Effects of cytokine combinations on CD8+ T-cell proliferation and function. Bulk ascites cells were stimulated with plate bound α-CD3 in media or 100% autologous ascites fluid for 48 h. Proliferation and function of CD8+ T cells in the presence of media, autologous ascites fluid (top panel), or ascites fluid in the presence of IL-2+ IL-12 (middle panel), or IL-2+ IL-12+ IL-21 (bottom panel) was assessed by measuring expression of Ki-67, CD107a, CCL4, IFN-γ, TNF-α, and IL-2 by flow cytometry. Data has been normalized to α-CD3 stimulated cells in media (i.e., media values were subtracted from each stimulation condition). *There was a significant effect of the cytokine combination for enhancing the indicated function compared to cells stimulated in media (Wilcoxon matched pairs t test, p<0.05).

Reasoning that a broader degree of functional enhancement might be achieved by simultaneously activating multiple STAT pathways, we assessed two combinations of cytokines: a) IL-2+ IL-12, and b) IL-2+ IL-12+ IL-21. Importantly, the latter combination would theoretically activate all STAT pathways relevant to CD8+ T-cell responses (i.e., STAT1, STAT3, STAT4, and STAT5). In general, both cytokine combinations induced T-cell proliferation similar to that seen with IL-2 (compare Fig. 2 with Fig. 1B). Notably however, CD8+ T cells from IROC028, which failed to proliferate in response to any single cytokine (Fig. 1B), proliferated in response to both cytokine combinations (Fig. 2). Moreover, both cytokine combinations were modestly more effective than single cytokines at enhancing IFN-γ and TNF-α production (Fig. 2). Despite this, neither cytokine combination enhanced expression of CD107a or IL-2 (Fig. 2). Similar results were seen with CD4+ T cells, although the effects of cytokines were generally weaker (data not shown). In summary, the addition of cytokine combinations engaging a wide range of STAT signaling pathways failed to fully restore T-cell function in the EOC ascites environment.

**Figure 3:**
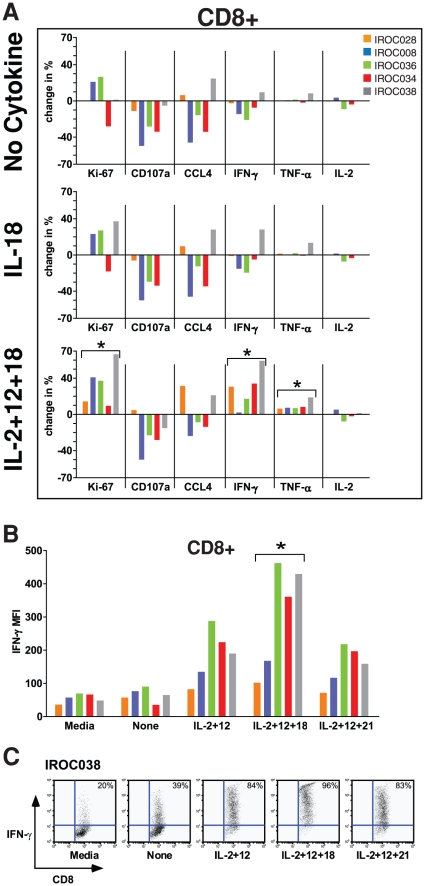
Effects of IL-18 alone, or in combination with IL-2 and IL-12, on CD8+ T-cell proliferation and function. Bulk ascites cells were stimulated with plate bound α-CD3 in media or 100% autologous ascites fluid for 48 h. Proliferation and function of CD8+ T cells in the presence of media or autologous ascites fluid (top panel) was assessed by measuring expression of Ki-67, CD107a, CCL4, IFN-γ, TNF-α, and IL-2 by flow cytometry. (**A**) Effect of IL-18 (middle panel) and IL-2+ IL-12+ IL-18 (bottom panel) on CD8+ T-cell proliferation and various functions in the presence of ascites fluid. Data has been normalized to α-CD3 stimulated cells in media (i.e., media values were subtracted from each stimulation condition). *There was a significant effect of IL-2+ IL-12+ IL-18 for enhancing the indicated function compared to cells stimulated in media (Wilcoxon matched pairs t test, p<0.05). (**B**) Mean fluorescence intensity (MFI) of IFN-γ positive CD8+ T cells was determined by intracellular IFN-γ staining. *The effect of IL-2+ IL-12+ IL-18 was significantly greater than the other two cytokine combinations (Wilcoxon matched pairs t test, p<0.05). (**C**) Representative flow cytometry plots demonstrating the effect of cytokine combinations on IFN-γ production on a per-cell basis. Shown are the mean flourescence intensity (Y axis) of IFN-γ production and the percentage of CD8+ cells expressing IFN-γ (numbers). All data are gated on CD8+ lymphocytes.

Finally, we assessed whether activation of non-STAT signaling pathways could further enhance T-cell function. To this end, we evaluated the cytokine IL-18, which belongs to the IL-1 cytokine family, activates the MyD88/NFκb pathway, and can directly enhance CD8+ T-cell responses in a variety of settings [39], [40], [41]. IL-18 alone had weak effects on T-cell proliferation and function (Fig. 3A). However, when combined with IL-2+ IL-12, IL-18 significantly enhanced expression of IFN-γ and, to a lesser extent, TNF-α (Fig. 3A). Moreover, the combination of IL-2+ IL-12+ IL-18 significantly increased the amount of IFN-γ produced on a per-cell basis, as evidenced by increased mean fluorescence intensity (MFI) (Fig. 3B, C). Nonetheless, the combination of IL-2+ IL-12+ IL-18 was unable to significantly enhance expression of CD107a or IL-2 (Fig. 3A) and only modestly enhanced granzyme B release (Supp. Fig. S2A). Likewise, this combination failed to restore expression of IL-1β, IL-1ra, IL-9, IL-17, G-CSF, GM-CSF, Mip-1α, PDGF-bb, or bFGF in culture supernatants (Supp. Fig. S2B). This combination did, however, weakly increase the levels of IL-5 and IL-10 in culture supernatants (Supp. Fig. S2C). Thus, the combination of IL-2+ IL-12+ IL-18 only partially restores T-cell functionality in the EOC environment.

### Exogenous cytokines induce new polyfunctional T-cell profiles

**Figure 4:**
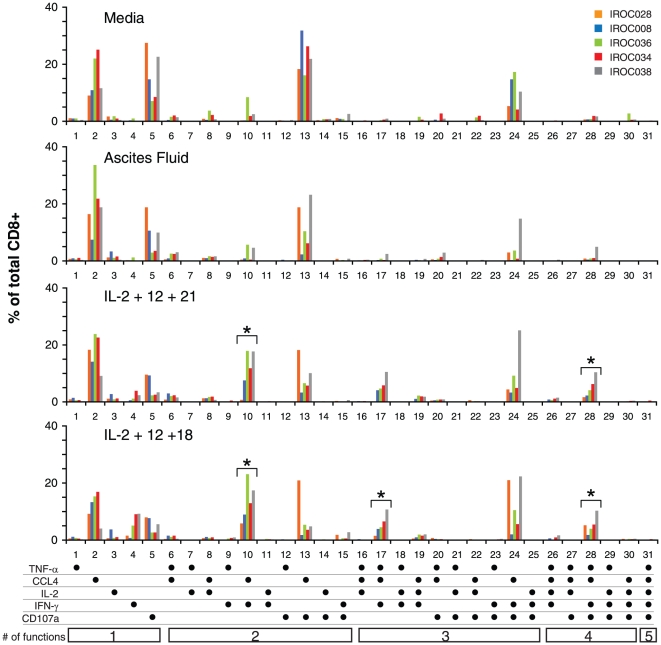
Effects of cytokine combinations on polyfunctional CD8+ T-cell responses. Bulk ascites cells were stimulated with plate bound α-CD3 in media or 100% autologous ascites fluid for 48 h in the presence or absence of the indicated cytokine combination. Boolean gate analysis was performed to quantify the number of T cells expressing each of 31 possible functional permutations. Shown are the results for CD8+ T cells stimulated in media, ascites fluid, or ascites fluid supplemented with the cytokine combinations IL-2+ IL-12+ IL-21 or IL-2+ IL-12+ IL-18. The frequency of T cells expressing a given permutation is expressed as a percentage of total CD8+ T cells. *For the indicated permutation, the effect of the cytokine combination was significantly greater than that seen with media (Wilcoxon matched pairs t test, p<0.05).

In theory, the enhanced activity of cytokine combinations compared to single cytokines could reflect either (a) different cytokines stimulating distinct subpopulations of T cells, or (b) cytokines acting synergistically to induce single T cells to perform multiple functions (i.e., induction of polyfunctional T cells). To investigate this issue, we used Boolean gate analysis to study the different functional permutations displayed by CD8+ and CD4+ T cells under different stimulation conditions. As seen in the top panel of Fig. 4, when bulk ascites cells were stimulated in complete media, only five predominant functional permutations were seen among CD8+ T cells: (a) non-functional (i.e., zero function) T cells (data not shown); (b) mono-functional T cells expressing CCL4 or CD107a (permutations 2 and 5), (c) bi-functional T cells expressing both CD107a and CCL4 (permutation 13), and (d) tri-functional T cells expressing CD107a, CCL4, and IFN-γ (permutation 24). In the presence of 100% ascites fluid, the number of non-functional T cells increased, whereas the other functional permutations were reduced with the exception of mono-functional T cells expressing CCL4 (Fig. 4, second panel). In general, addition of single cytokines failed to significantly change the functional permutations seen with ascites alone, although IL-2 had weak effects on several permutations (Supp. Fig. S3). In contrast, the addition of cytokine combinations (either IL-2+ IL-12+ IL-21, or IL-2+ IL-12+ IL-18) markedly decreased the number of zero- and monofunctional T cells and generated three new polyfunctional permutations: a) CCL4 and IFN-γ (permutation 10); b) CCL4, IFN-γ, and TNF-α (permutation 17); and c) CCL4, IFN-γ, TNF-α, and CD107a (permutation 28) (Fig. 4, third and fourth panels). Similar trends were seen for CD4+ T cells, although the effects were generally weaker (data not shown). Thus, these combinations of cytokines appear to act synergistically to induce polyfunctional responses by individual T cells as opposed to stimulating multiple subpopulations of mono-functional T cells.

## Discussion

We show here that the ascites environment in human EOC has variable effects on CD8+ and CD4+ T-cell proliferation and indeed can even enhance proliferation in some cases. By contrast, ascites generally inhibits expression of IFN-γ, TNF-α, CCL4, and CD107a by T cells, resulting in a preponderance of zero- or monofunctional T cells. Moreover, the ascites environment generally inhibited expression of IL-1β, IL-1ra, IL-9, IL-17, G-CSF, GM-CSF, Mip-1α, PDGF-bb, and bFGF in culture supernatants. Although IL-2 could restore T-cell proliferation, it was largely ineffective at enhancing other T-cell functions, as were other single agent cytokines (IL-12, IL-18, and IL-21). Combinations of cytokines that activate complementary signaling pathways (in particular IL-2+ IL-12+ IL-18) were able to enhance expression of IFN-γ, TNF-α, and CCL4 but had negligible effects on other T-cell functions. Thus, exogenous cytokines can partly restore T-cell function in the human EOC environment; however, alternative strategies will be required to fully restore T-cell function.

One limitation of this study is the relatively small sample size, which was necessary given the number of cytokines and functional read-outs involved. Despite the small sample size, there was a notable degree of similarity between the overall functional profiles of all five patient samples, both at baseline and after cytokine stimulation. This suggests that the diverse immunosuppressive mechanisms present in ovarian cancer might ultimately converge toward a common T-cell functional state governed by a conserved set of regulatory signals.

The release of cytotoxic granules is a major mechanism by which T cells kill virus-infected and transformed cells [42]. We found that ascites fluid strongly inhibited T cell degranulation, and this was only partly restored by exogenous cytokines (as evidenced by modest enhancement of granzyme B release). Previous studies have shown that the combination of IL-2 with TCR stimulation [9] or IL-12 [10], [11] could fully restore LAK cytotoxicity in the presence of 50% ascites fluid. This discrepancy with our results may reflect the more demanding culture conditions we employed, in that T cells were assayed in the presence of 100% ascites fluid and a full complement of other tumor-associated cell types.

The lack of IL-2 production by both CD8+ and CD4+ T cells in these experiments is also consistent with an impaired anti-tumor response. In our mouse model of ovarian cancer [8], tumor regression was entirely dependent on IL-2/IL-15 signaling. Likewise, Gattinoni and colleagues demonstrated that effector CD8+ T cells capable of potent *in vitro* anti-tumor cytotoxicity and IFN-γ production, but not IL-2 production, had limited anti-tumor activity *in vivo*, whereas T cells that had low *in vitro* cytotoxicity, but high IL-2 production, mediated superior anti-tumor responses [12]. As CD8+ T cells generally lose the ability to produce IL-2 upon differentiation, the failure of cytokines to enhance IL-2 production suggests that most tumor-associated T cells may be terminally differentiated effectors [13]. If so, future efforts should be directed toward isolating and expanding less differentiated subsets of T cells with greater pluripotency.

In addition to failed IL-2 production, we found that ascites fluid inhibited the expression of numerous other cytokines (IL-1β, IL-1ra, IL-9, IL-17, G-CSF, GM-CSF, Mip-1α, PDGF-bb, and bFGF) in culture supernatants. Although the multiplex cytokine assay we employed cannot discriminate which cell type(s) is inhibited from producing these cytokines, many of these cytokines have documented roles in T-cell responses and hence their reduced levels are expected to impact T-cell function. In particular, IL-17 is produced by the Th17 subset of CD4+ T cells under inflammatory conditions and has been correlated with improved overall survival in EOC [5]. Given that the combination of IL-2+ IL-12+ IL-18 failed to enhance IL-17 production, alternative strategies will be required to promote this effector pathway within the tumor environment. For example, IL-1β, IL-6, TGF-β, IL-23, and ICOS have all been shown to influence the differentiation, expansion or function of Th17 cells [4], [43], and thus could potentially be manipulated to promote IL-17 production *in vivo*.

In contrast to these other cytokine defects, the combination of IL-2+ IL-12+ IL-18 potently enhanced IFN-γ production by CD8+ T cells (Fig. 3B, C). IFN-γ plays a central role in anti-tumor immunity by inhibiting tumor proliferation and angiogenesis, and upregulating tumor antigen presentation. Endogenous IFN-γ protects against tumor development and, in a number of tumor models, is critical for anti-tumor immunity [44]. Moreover, IFN-γ and IFN-γ receptor expression, as well as several genes downstream of IFN-γ, are associated with increased survival in human EOC [1]. How might IL-2, IL-12, and IL-18 cooperatively enhance IFN-γ production? First, these cytokines reciprocally upregulate expression of their respective receptors, resulting in enhanced sensitivity of T cells to all three cytokines [45], [46], [47], [48]. Second, these cytokines can mediate synergistic and complementary signaling events [37], [49], [50]. For example, IL-2 and IL-12 synergistically activate the p38 MAPK pathway to enhance IFN-γ expression [51]. Likewise, IL-12 and IL-18 activate the transcription factors STAT4 and AP-1, respectively, which can synergistically enhance IFN-γ promoter activity [52]. Thus, the coordinated activation of both STAT and non-STAT signaling pathways appears important for maximal expression of IFN-γ. It is noteworthy, however, that these signals were not sufficient to restore expression of many other cytokines. This suggests that, in future, cytokines other than IFN-γ should be used as read-outs if one wishes to optimize the full range of T cell functions for cancer immunotherapy.

The combination of IL-2+ IL-12+ IL-18 also induced significant changes in polyfunctional T-cell permutations. Although the clinical significance of these polyfunctional profiles has yet to be determined for EOC, insights can be gained from other disease settings. Two of the four functional permutations seen at baseline (specifically, monofunctional CD107a, and bi-functional CD107a and CCL4; Fig. 4) are also elevated in CD8+ T cells from HIV progressors compared to non-progressors, suggesting that these permutations may represent functionally exhausted T cells [19], [20]. Addition of IL-2+ IL-12+ IL-18 decreased the number of CD8+ T cells exhibiting these “exhausted” functional permutations. Moreover, it caused the emergence of three new polyfunctional permutations: a) CCL4 and IFN-γ; b) CCL4, IFN-γ, and TNF-α; and c) CCL4, IFN-γ, TNF-α, and CD107a (Fig. 4). Notably, these three permutations were shown to be induced in CD8+ T cells by a protective vaccine against smallpox [14]. Furthermore, tetra-functional (CCL4, IFN-γ, TNF-α, and CD107a) CD8+ T cells are found at significantly higher frequencies in HIV long-term non-progressors compared to progressors [19], [20]. Finally, higher proportions of tumor-reactive T cells co-expressing CCL4, IFN-γ, and TNF-α were detected in melanoma patients with favorable clinical responses to α-CTLA-4 treatment [34]. These prior reports suggest that the polyfunctional permutations induced by IL-2+ IL-12+ IL-18 may have therapeutic benefit in EOC, although this suggestion needs to be validated *in vivo*.

Our findings shed new light on previous clinical trials in EOC involving IL-2 and IL-12. Intraperitoneal administration of IL-2 resulted in an approximately 25% overall response rate in two studies [53], [54], while IL-2 in combination with retinoic acid as a maintenance therapy demonstrated a relatively modest, but statistically significant, prolongation of progression-free and overall survival in a phase II trial [55], [56]. By contrast, IL-12 has shown little therapeutic efficacy in EOC in two clinical trials [57], [58]. The limited efficacy of IL-2 and IL-12 as monotherapies is consistent with our results demonstrating the modest functional activity of these cytokines as single agents. Our results would also predict that IL-18 and IL-21 may have limited clinical efficacy when used as monotherapies. Indeed, although IL-18 and IL-21 have not been evaluated in EOC patients, these cytokines as single agents showed limited clinical benefit in the setting of metastatic melanoma [59], [60], [61], [62], [63], [64]. When combined, IL-2, IL-12, and IL-18 showed the most promise in the *in vitro* experiments reported here, but unfortunately these cytokines would likely have significant side effects if given in combination to patients [47], [65], [66]. While it may be possible to reduce toxicity by optimizing dose and route of delivery (e.g., intraperitoneal administration) [47], [67], one is still left with the fact that this combination does not fully restore T-cell function, as manifested by impaired expression of CD107a, IL-2, and other cytokines. Thus, our findings indicate that alternative therapeutic strategies involving other signaling pathways will be required to unleash the full potential of host T-cell responses against EOC.

## Materials and Methods

### Study subjects and specimens

#### Ethics Statement

Newly diagnosed patients with high-grade serous EOC gave written informed consent under protocols approved by the Research Ethics Board of the BC Cancer Agency and the University of British Columbia.

Tumor tissue and ascites were obtained during primary surgery prior to any other treatment. Ascites was centrifuged at 300 g, and supernatants (ascites fluid) were stored at −80°C. Ascites cell (AC) pellets containing large quantities of red blood cells were treated with ACK lysis buffer. AC pellets were cryopreserved in liquid nitrogen. Upon thawing, ascites cells were rested in complete RPMI (RPMI 1640 with 10% FBS, 25 mM HEPES, 1 mM sodium pyruvate, 2 mM L-glutamine, and 50 µm β-mercaptoethanol) for 4 h at 37°C prior to experiments.

### Antibodies, cytokines, and ELISAs

Flow cytometry was performed with the following fluorochrome-conjugated antibodies (BD Biosciences): CD3 (FITC, PECy5), CD4 (FITC, PE), CD8 (PECy5, APC-H7), CD14 (PE), CD19 (PE), CD25 (PECy5), CD56 (PE), CD107a (PECy5), Ki-67 (FITC), CCL4 (Mip-1β, PE), IL-2 (APC), TNF-α (PECy7), and IFN-γ (PE, AlexaFluor 700). Foxp3 (PE) was from eBioscience. Recombinant human IL-12, IL-21 (both from Peprotech), and IL-18 (R&D Systems) were used at 100 ng/ml. IL-2 (Proleukin) was used at 100 U/ml. ELISA was used to measure TGF-β (latent and active; eBioscience) and granzyme B (Mabtech) in ascites fluid, according to manufacturers' instructions.

### Proliferation assays

Ascites cells (AC) were seeded in triplicate in 96-well flat bottom plates at 1×10^5^ cells per well. AC were resuspended in media or 100% ascites fluid and were left unstimulated or stimulated with plate bound α-CD3ε antibody (clone OKT3, eBioscience) previously coated at 2.5 µg/ml for 2 h at 37°C. Cells were stimulated for 48 h in the presence or absence of cytokines, and T-cell proliferation was measured by detection of Ki-67 using flow cytometry. Cells were washed with FACS buffer (1% FBS in PBS), permeabilized with ice-cold 100% methanol and incubated at −20°C for at least 1 h. Cells were then washed with FACS buffer and triple-stained with pre-titered antibodies to Ki-67, CD4, and CD8 for at least 30 min at room temperature in the dark. Cells were washed and analyzed with a FACSCalibur flow cytometer (Becton Dickinson). Data was analyzed using FlowJo software (Tree Star Inc.).

### Assessment of T-cell functional markers

Bulk ascites cells were washed with serum-free RPMI, resuspended at 5×10^5^ cells/ml in complete RPMI or autologous ascites fluid, and plated in 48-well plates at 3×10^5^ cells/well with or without plate bound α-CD3ε. Cytokines and α-CD107a antibody were added to appropriate wells, and cells were incubated for 48 h at 37°C. As per standard protocol, α-CD107a antibody was added during stimulation because CD107a, which is associated with the membranes of cytotoxic granules, transiently localizes to the surface of T cells undergoing cellular degranulation [68]. Thus, cell-surface expression of CD107a serves as a surrogate marker of cytotoxicity. As a positive control, PMA plus ionomycin (both from Sigma) were used at 40 ng/ml and 1.5 µg/ml, respectively. GolgiStop (BD Bioscience, 1∶1500 dilution) and brefeldinA (Sigma, 5 µg/ml) were added for the final 6 h of culture. Cells were harvested, labeled with α-CD4 and CD8 antibodies, fixed and permeabilized with Cytofix/Cytoperm buffer (BD Bioscience) according to manufacturer's instructions, washed with Perm/Wash buffer, and stored in FACS buffer overnight at 4°C. Cells were washed again with Perm/Wash buffer, and intracellular staining was performed using antibodies against CCL4, IL-2, TNF-α, and IFN-γ. Cells were fixed with 2% formaldehyde and stored in FACS buffer overnight at 4°C. Samples were analyzed with a BD Bioscience FACSVantage DIVA modified with the Octagon array. SSC Area vs. SSC W were used to gate out doublets. Data was analyzed with FlowJo software (TreeStar) and exported to PESTLE v1.6.1 (Mario Roederer, NIH) for further data analysis.

### Multiplex cytokine assays

Supernatants harvested from cultures described in “Proliferation assays” were assessed for expression of 27 cytokines using the Bio-Plex human cytokine 27-plex panel (Bio-Rad) according to manufacturer's recommended protocol. The evaluated cytokines included interleukins-1β, -1ra, -2, -4, -5, -6, -7, -8, -9, -10, -12 (p70), -13, -15, and -17, and basic FGF, eotaxin, G-CSF, GM-CSF, IFN-γ, IP-10, MCP-1, CCL3/Mip-1α, CCL4, PDGF-BB, RANTES, TNF-α, and VEGF.

## Supporting Information

Figure S1 **Impact of ascites fluid on granzyme B and cytokine expression.** Bulk ascites cells were stimulated for 48 h with plate bound α-CD3 in media or 100% autologous ascites fluid. The expression of **(A)** granzyme B, and **(B)** 27 cytokines was assessed in culture supernatants using ELISA and multiplex cytokine assays, respectively. Data is shown for all α-CD3-induced cytokines that were significantly impaired by ascites fluid (Wilcoxon matched pairs t test, p<0.05). * Indicates maximum discernible concentration; actual value may exceed this value.
(EPS)

Figure S2 **Effects of the cytokine combination of IL-2+ IL-12+ IL-18 on granzyme B and cytokine expression.** Bulk ascites cells were stimulated for 48 h with plate bound α-CD3 in media or 100% autologous ascites fluid in the absence or presence of IL-2+ IL-12+ IL-18. The expression of **(A)** granzyme B, and **(B)** the indicated cytokines was assessed in culture supernatants using ELISA and multiplex cytokine assays, respectively. Data was normalized to α-CD3 stimulated cells in media by calculating the % change using the following equation: (B – A)/A, where B is the measured value in the indicated condition and A is the measured value in media. * IL-2+ IL-12+ IL-18 significantly enhanced the indicated function compared to cells stimulated in ascites fluid alone (Wilcoxon matched pairs t test, p<0.05).
(EPS)

Figure S3 **Effects of IL-2, IL-12, IL-18, and IL-21 on polyfunctional CD8+ T-cell responses.** Bulk ascites cells were stimulated with plate bound α-CD3 in media or 100% autologous ascites fluid for 48 h in the presence or absence of the indicated cytokine. Boolean gate analysis was performed to quantify the number of T cells expressing each of 31 possible functional permutations. Shown are the results for CD8+ T cells stimulated in ascites fluid in the presence of IL-2, IL-12, IL-18, or IL-21. The frequency of T cells expressing a given permutation is expressed as a percentage of total CD8+ T cells. *For the indicated permutation, the effect of the cytokine was significantly greater than that seen with media (Wilcoxon matched pairs t test, p<0.05).
(EPS)
